# The Prevalence of COVID-19 Vaccination and Vaccine Hesitancy in Pregnant Women: An Internet-based Cross-sectional Study in Japan

**DOI:** 10.2188/jea.JE20210458

**Published:** 2022-04-05

**Authors:** Yoshihiko Hosokawa, Sumiyo Okawa, Ai Hori, Naho Morisaki, Yoko Takahashi, Takeo Fujiwara, Shoji F. Nakayama, Hiromi Hamada, Toyomi Satoh, Takahiro Tabuchi

**Affiliations:** 1 Department of Obstetrics and Gynecology, Faculty of Medicine, University of Tsukuba, Ibaraki, Japan; 2 Institute for Global Health Policy Research, Bureau of International Health Cooperation, National Center for Global Health and Medicine, Tokyo, Japan; 3 Department of Global Public Health, Faculty of Medicine, University of Tsukuba, Ibaraki, Japan; 4 Department of Social Medicine, National Center for Child Health and Development, Tokyo, Japan; 5 Center for Postgraduate Education and Training, National Center for Child Health and Development, Tokyo, Japan; 6 Department of Global Health Promotion, Graduate School of Medical and Dental Sciences, Tokyo Medical and Dental University, Tokyo, Japan; 7 Japan Environment and Children’s Study Programme Office, National Institute for Environmental Studies, Ibaraki, Japan; 8 Cancer Control Center, Osaka International Cancer Institute, Osaka, Japan

**Keywords:** COVID-19, vaccine, vaccine hesitancy, pregnancy

## Abstract

**Background:**

Reluctance of people to receive recommended vaccines is a growing concern, as distribution of vaccines is considered critical to ending the COVID-19 pandemic. There is little information regarding pregnant women’s views toward coronavirus vaccination in Japan. Therefore, we investigated the vaccination rate and reasons for vaccination and vaccine hesitancy among pregnant women in Japan.

**Methods:**

We conducted a cross-sectional study involving 1,791 pregnant women using data from the Japan “COVID-19 and Society” Internet Survey, conducted from July to August 2021, and valid response from 1,621 respondents were analyzed. We defined participants with vaccine hesitancy as those who identified with the statement “I do not want to be vaccinated” or “I want to ‘wait and see’ before getting vaccinated.” Multivariate Poisson regression analysis was used to investigate the factors contributing to vaccine hesitancy.

**Results:**

The prevalence of vaccination and vaccine hesitancy among pregnant women was 13.4% (*n* = 217) and 50.9% (*n* = 825), respectively. The main reasons for hesitancy were concerns about adverse reactions and negative effects on the fetus and breastfeeding. Vaccine hesitancy was significantly associated with the lack of trust in the government (adjusted prevalence ratio, 1.26; 95% confidence interval, 1.03–1.54). Other factors, such as age, educational attainment, and state of emergency declaration, were not associated with vaccine hesitancy.

**Conclusions:**

COVID-19 vaccination is not widespread among pregnant women in Japan, although many vaccines have been shown to be safe in pregnancy. Accurate information dissemination and boosting trust in the government may be important to address vaccine hesitancy among pregnant women.

## INTRODUCTION

The spread of coronavirus disease 2019 (COVID-19) is a serious problem worldwide, including in Japan. Vaccination is considered an effective preventive strategy for COVID-19 control, in addition to other infection prevention measures.^1^ In Japan, COVID-19 vaccination has been conducted, with priority given to the elderly and populations with existing underlying diseases.^2^^,^^3^ Recently, pregnancy has been recognized as a risk factor for severe COVID-19 infection, and COVID-19 infection during pregnancy is associated with poor maternal and neonatal outcomes.^4^^,^^5^ Due to the demonstrated safety of COVID-19 vaccination during pregnancy,^6^^–^^8^ the Centers for Disease Control and Prevention in the United States recommends COVID-19 vaccination for all pregnant women.^9^

In Japan, vaccination during pregnancy is similarly recommended.^10^ The Japan Society of Obstetrics and Gynecology initially published recommendations that pregnant women should not be excluded from vaccination, but vaccination should be avoided until 12 weeks of gestation.^10^ However, based on studies regarding vaccination during pregnancy,^6^^–^^8^ the statement on vaccine avoidance until 12 weeks gestation was removed in June 2021^11^ and changed to a statement recommending vaccination in all trimesters in August 2021.^12^

Current vaccination statistics in Japan do not indicate the number of women who have been vaccinated during pregnancy.^13^ Previous studies have reported rates of vaccine hesitancy among women, younger people, and younger women.^14^^,^^15^ According to a study conducted by Yoda et al in September 2020, the percentage of vaccine hesitancy was 36.8% among women and 32.0% among men, where the participants who responded that ‘they did not want to be vaccinated’ and ‘were not sure whether they wanted to be vaccinated’ were considered as exhibiting vaccine hesitancy.^15^ The percentage of vaccine hesitancy was 34.6% in the population aged 20–59 years and 28.1% in the population aged 60 years and older. According to a study conducted by Okubo et al in February 2021, vaccine hesitancy, exhibited in participants who did not want to be vaccinated, was 15.6% among women under 40 years of age, 14.2% among men under 40 years of age, and 13.2% among women aged 40–64 years.^14^ However, no study has focused on vaccine hesitancy specifically among pregnant women. Considering the widespread use of vaccination during pregnancy, it is necessary to assess the current level of vaccine coverage. In addition, it is important to determine the rate of vaccine hesitancy in pregnant women and their main concerns regarding vaccination. Furthermore, identification of factors associated with vaccine hesitancy is essential in developing more effective strategies for vaccine dissemination.

Therefore, we aimed to investigate the rate of COVID-19 vaccination in pregnant women in Japan and evaluate pregnant women’s reasons for being vaccinated. We also aimed to determine the reasons for hesitancy among those unvaccinated.

## METHODS

### Data setting

This was a cross-sectional internet-based survey conducted as part of the Japan COVID-19 and Society Internet Survey (JACSIS).^16^ The study samples for each survey were retrieved from the pooled panels of an internet research agency.^17^ First, a screening survey was conducted on July 24, 2021 to determine the eligibility of participants who were pregnant and expected to give birth by December 2021, and 2,425 participants were determined to be eligible for this study. Next, the internet research agency distributed a questionnaire to all eligible pregnant women through an email with a designated website. Data on current pregnancies were collected between July 28 and August 30, 2021. Data were collected from 1,791 women (response rate, 73.9%), and a total of 1,621 of these women were included in the analysis, following the exclusion of 170 women who provided irrelevant or contradictory information. This method mirrored those performed in previous studies by the same research group.^14^^,^^16^ The prefectures where the selected pregnant women lived were representative of all prefectures in Japan (eTable 1).

As an incentive for study participation, the participants received credit points called “Epoints,” which can be used for online shopping and cash conversion. Although the exact value of each Epoint was not disclosed under the Internet survey agency’s rule, 1 Epoint was assumed to be equivalent to around 100 Japanese yen (JPY; approximately 1 United States dollar).

### Definitions of “vaccinated against COVID-19” and “COVID-19 vaccine hesitancy”

The survey was designed so that participants had to answer the question, “What is your opinion on vaccination against the new coronavirus infections?” Participants answered by choosing one of the following options: “I have already been vaccinated,” “I want to be vaccinated,” “I want to ‘wait and see’ before getting the vaccine,” and “I do not want to be vaccinated.” Since the number of vaccinations was not included in the survey, participants who claimed that they had already been vaccinated were defined as those who had received at least one dose of the COVID-19 vaccine. Additionally, those who answered “I have already been vaccinated” or “I want to be vaccinated” were defined as the “Acceptant” group, while those who answered “I want to ‘wait and see’ before getting the vaccine” or “I do not want to be vaccinated” were defined as the “Hesitant” group.^18^

Participants in the Acceptant group were asked why they wanted to get vaccinated; similarly, those in the Hesitant group who answered “I want to ‘wait and see’ before getting the vaccine” were asked why they wanted to get vaccinated and why they wanted to delay their vaccination. Participants who answered “I do not want to be vaccinated” were asked why they were hesitant to get vaccinated. Multiple responses from a predetermined list of options were allowed; therefore, the total percentage for each reason did not necessarily add up to 100%.

### Potential factors associated with vaccine hesitancy

The covariates were set in line with previous studies.^14^^,^^15^ We identified potential risk factors for vaccine hesitancy as follows: maternal age, gestational age (<28 weeks, 28–31 weeks, 32–36 weeks, or ≥37 weeks), pre-pregnancy body mass index (<18.5, 18.5–24.9, 25.0–29.9 or ≥30 kg/m^2^), use of in vitro fertilization (yes or no), active smoker status (never, former, or current), daily passive smoking status (yes or no), alcohol consumer status (never, former, or current), level of educational attainment (≤12 years or ≥13 years), marital status (married or other), household income in JPY (<5 million JPY, 5 to <8 million JPY, or ≥8 million JPY), working status during the survey (working, not working with maternity leave, or not working without maternity leave), and comorbidities (chronic hypertension, diabetes mellitus, asthma, thyroid disorders, chronic kidney disease, or autoimmune disease). We also included current or previous COVID-19 infection (yes or no) and level of fear of COVID-19 (scores ranging from 7 to 35 using the Fear of COVID-19 Scale) as factors.^19^^,^^20^ Additionally, we divided all prefectures where participants lived into three groups according to their state of emergency declaration (none, semi-emergency, or emergency). The declaration of a state of emergency was issued by the government to each prefecture where the COVID-19 pandemic was spreading to control its rapid spread. Businesses, restaurants, and residents were requested to prevent the spread of the infection. The emergency requests included preventive measures, such as restricting the organization of events and shortening of business hours. These requests did not include any penalties in contrast to other countries with pandemic. Since the declaration of emergency changed daily (eTable 2), the definition was based on the state declared on the date of response. For trust in the government, participants were categorized into groups (trust or mistrust). Participants who answered “yes” or “somewhat yes” to the question, “Is the government trustworthy?” were defined as having trust in the government, while those who answered “no” or “not so much” were defined as having mistrust in the government.^21^ We also included the response period (07/28–08/20, 08/21–08/24, or 08/25–08/31) as a potential factor influencing a respondent’s intention to get vaccinated, given that on August 19, 2021, the news reported that in Japan, a pregnant woman with COVID-19 went into preterm labor but was unable to find a hospital that could manage her; the woman delivered at home, which resulted in the death of her newborn.^22^

### Statistical analysis

Descriptive statistics were calculated for the overall population and Acceptant and Hesitant groups. Chi-squared tests were used to compare the difference between vaccine hesitancy rates according to each potential risk factor. Poisson regression models were used to calculate the prevalence ratios (PRs) and 95% confidence intervals (CIs) for vaccine hesitancy, adjusting for the covariates described previously, because the outcome was more than 10%.^23^

IBM SPSS version 26.0 (IBM Japan, Ltd, Tokyo, Japan) was used for all statistical analyses. A two-sided *P*-value of less than 0.05 was considered to indicate statistical significance.

### Ethics approval

This study was approved by the Bioethics Review Committee of Osaka International Cancer Institute, Japan. All procedures in this study were performed in accordance with the ethical guidelines for medical and health research involving human subjects enforced by the Japanese government’s Ministry of Health, Labour, and Welfare, and with the 1964 Helsinki Declaration and its later amendments. Informed consent was obtained electronically, and all participants were informed of their right to withdraw from the study.

## RESULTS

Of the 1,621 participants, 13.4% (*n* = 217) had received at least one COVID-19 vaccine (Table 1). The Hesitant group comprised 50.9% (*n* = 825) of the study population and the Acceptant group comprised 49.1% (*n* = 796) (Table 1).

**Table 1.** Demographic and medical characteristics of pregnant women included in the analysis

	All pregnant women (*n* = 1,621)	COVID-19 vaccine intention			

		Already vaccinated (*n* = 217)	Want to be vaccinated (*n* = 579)	Want to “wait and see” before getting vaccinated (*n* = 689)	Do not want to be vaccinated (*n* = 136)
Response period (2021), *n* (%)					
7/28–8/20	184 (11.4)	17 (7.8)	66 (11.4)	78 (11.3)	23 (16.9)
8/21–8/24	987 (60.9)	129 (59.4)	355 (61.3)	420 (61.0)	83 (61.0)
8/25–8/31	450 (27.8)	71 (32.7)	158 (27.3)	191 (27.7)	30 (22.1)
Maternal age at response, *n* (%)					
<25 years	72 (4.4)	4 (1.8)	23 (4.0)	39 (5.7)	6 (4.4)
25–29 years	505 (31.2)	61 (28.1)	166 (28.7)	230 (33.4)	48 (35.3)
30–34 years	629 (38.8)	94 (43.3)	239 (41.3)	240 (34.8)	56 (41.2)
35–39 years	340 (21.0)	54 (24.9)	122 (21.1)	143 (20.8)	21 (15.4)
≥40 years	75 (4.6)	4 (1.8)	29 (5.0)	37 (5.4)	5 (3.7)
Nulliparity, *n* (%)	868 (53.5)	131 (60.4)	274 (47.3)	385 (55.9)	78 (57.4)
Pre-pregnancy BMI, kg/m^2^, *n* (%)					
<18.5	326 (20.1)	39 (18.0)	111 (19.2)	145 (21.0)	31 (22.8)
18.5–24.9	1,165 (71.9)	162 (74.7)	429 (74.1)	479 (69.5)	95 (69.9)
25–29.9	102 (6.3)	11 (5.1)	31 (5.4)	53 (7.7)	7 (5.1)
≥30	28 (1.7)	5 (2.3)	8 (1.4)	12 (1.7)	3 (2.2)
In vitro fertilization, *n* (%)	195 (12.0)	27 (12.4)	64 (1.4)	85 (12.3)	19 (14.0)
Smoking, *n* (%)					
Never	1,208 (74.5)	158 (72.8)	437 (75.5)	515 (74.7)	98 (72.1)
Former	380 (23.4)	54 (24.9)	132 (22.8)	162 (23.5)	32 (23.5)
Current	33 (2.0)	5 (2.3)	10 (1.7)	12 (1.7)	6 (4.4)
Alcohol drinking, *n* (%)					
Never	365 (22.5)	45 (20.7)	117 (20.2)	166 (24.1)	37 (27.2)
Former	1,194 (73.7)	159 (73.3)	438 (75.6)	503 (73.0)	94 (69.1)
Current	62 (3.8)	13 (6.0)	24 (4.1)	20 (2.9)	5 (3.7)
Gestational age, *n* (%)					
<28 weeks	356 (22.0)	65 (30.0)	119 (20.6)	130 (18.9)	42 (30.9)
28–31 weeks	285 (17.6)	43 (19.8)	97 (16.8)	118 (17.1)	27 (19.9)
32–36 weeks	468 (28.9)	63 (29.0)	156 (26.9)	211 (30.6)	38 (27.9)
≥37 weeks	512 (31.6)	46 (21.2)	207 (35.8)	230 (33.4)	29 (21.3)
Educational attainment ≥13 years, *n* (%)	1,369 (84.5)	200 (92.2)	498 (86.0)	558 (81.0)	113 (83.1)
Married, *n* (%)	1,590 (98.1)	214 (98.6)	574 (99.1)	670 (97.2)	132 (97.1)
Current or previous COVID-19, *n* (%)	14 (0.9)	2 (0.9)	5 (0.9)	5 (0.7)	2 (1.5)
Maternal complications, *n* (%)					
Chronic hypertension	45 (2.8)	8 (3.7)	14 (2.4)	20 (2.9)	3 (2.2)
Diabetes	59 (3.6)	8 (3.7)	16 (2.8)	28 (4.1)	7 (5.1)
Asthma	183 (11.3)	21 (9.7)	65 (11.2)	77 (11.2)	20 (14.7)
Thyroid disease	92 (5.7)	18 (8.3)	25 (4.3)	39 (5.7)	10 (7.4)
Depression	113 (7.0)	22 (10.1)	39 (6.7)	45 (6.5)	7 (5.1)
Chronic kidney disease	9 (0.6)	2 (0.9)	1 (0.2)	4 (0.6)	2 (1.5)
Autoimmune disease	19 (1.2)	2 (0.9)	4 (0.7)	8 (1.2)	5 (3.7)
Homeowner, *n* (%)	646 (39.9)	70 (32.3)	250 (43.2)	270 (39.2)	56 (41.2)
Household income per year, *n* (%)					
<5 million JPY	358 (22.1)	40 (18.4)	115 (19.9)	167 (24.2)	36 (26.5)
5 to <8 million JPY	491 (30.3)	43 (19.8)	183 (31.6)	226 (32.8)	39 (28.7)
≥8 million JPY	666 (41.1)	121 (55.8)	243 (42.0)	252 (36.6)	50 (36.8)
Declined to answer or do not know	106 (6.5)	13 (6.0)	38 (6.6)	44 (6.4)	11 (8.1)
Occupational status, *n* (%)					
Working	578 (35.7)	82 (37.8)	245 (42.3)	259 (37.6)	40 (29.4)
Not working with maternity leave	417 (25.7)	91 (41.9)	123 (21.2)	163 (23.7)	40 (29.4)
Not working without maternity leave	626 (38.6)	44 (20.3)	211 (36.4)	267 (38.8)	56 (41.2)
Work type, *n* (%)					
Desk work	711 (43.9)	115 (53.0)	264 (45.6)	284 (41.2)	48 (35.3)
Interpersonal	676 (41.7)	117 (53.9)	215 (37.1)	295 (42.8)	49 (36.0)
Physical work	340 (21.0)	52 (24.0)	110 (19.0)	153 (22.2)	25 (18.4)
Fear of COVID-19 scale, mean (SD)	20.0 (5.2)	19.8 (4.8)	20.2 (5.0)	20.3 (5.1)	18.3 (6.3)
Mistrust in the government, *n* (%)	1,355 (83.6)	170 (78.3)	470 (81.2)	591 (85.8)	124 (91.2)
Local state of emergency, *n* (%)					
None	264 (16.3)	22 (10.1)	87 (15.0)	132 (19.2)	23 (16.9)
Semi-emergency	417 (25.7)	60 (27.6)	157 (27.1)	164 (23.8)	36 (26.5)
Emergency	940 (58.0)	135 (62.2)	335 (57.9)	393 (57.0)	77 (56.6)
Total days of emergency declared until response date, median (range)	5 (0–100)	8 (0–94)	5 (0–90)	4 (0–100)	4 (0–94)

COVID-19 vaccine intention, *n* (%)					
Already vaccinated	217 (13.4)				
Want to be vaccinated	579 (35.7)				
Want to “wait and see” before getting vaccinated	689 (42.5)				
Do not want to be vaccinated	136 (8.4)				

BMI, body mass index; COVID-19, coronavirus disease 2019; JPY, Japanese yen; SD, standard deviation.

**Figure 1.**
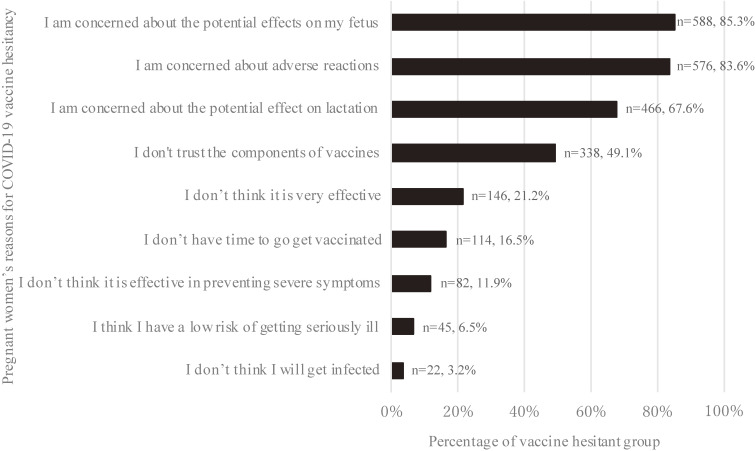
The reasons for COVID-19 vaccine hesitancy reported by pregnant women There were 689 participants who responded to the survey with the statement “I want to ‘wait and see’ before getting the vaccine.” The reasons participants gave for their vaccine hesitancy are listed in order of frequency. Participants could choose more than one reason; therefore, the total number of responses exceeded 100%.

The participants’ reasons for their acceptance to get vaccinated is presented in eTable 3. Among those who answered the survey with the statement “I want to ‘wait and see’ before getting the vaccine,” the major reasons for vaccine hesitancy were anxiety about potential negative effects on the fetus (85.3%, *n* = 588), adverse reactions at the time of injection (83.6%, *n* = 576), anxiety about potential negative effects on the breastfed infant (67.6%, *n* = 466), and the trustworthiness and reliability of the vaccine (49.1%, *n* = 338) (Figure 1).

Poisson regression analysis indicated that mistrust in the government was the only factor significantly associated with higher vaccine hesitancy, even after adjusting for other potential factors. The adjusted PR for vaccine hesitancy, given mistrust in the government, was 1.26 (95% CI, 1.03–1.54) (Table 2).

**Table 2.** Factors associated with vaccine hesitancy among all pregnant women in this study

	Acceptance (*n* = 796)		Hesitant (*n* = 825)		*P*-value	aPR	95% CI
Response period (2021)	*n*	%	*n*	%			
7/28–8/20	83	45.1%	101	54.9%	0.417	1.00	
8/21–8/24	484	49.0%	503	51.0%		0.99	(0.79–1.25)
8/25–8/31	229	50.9%	221	49.1%		0.96	(0.74–1.23)
Gestational age							
<28 weeks	184	51.7%	172	48.3%	0.579	1.00	
28–31 weeks	140	49.1%	145	50.9%		0.97	(0.81–1.17)
32–36 weeks	219	46.8%	249	53.2%		0.91	(0.73–1.15)
≥37 weeks	253	49.4%	259	50.6%		0.89	(0.70–1.13)
Maternal age							
<25 years	27	37.5%	45	62.5%	0.012	1.20	(0.86–1.67)
25–29 years	227	45.0%	278	55.0%		1.15	(0.98–1.36)
30–34 years	333	52.9%	296	47.1%		1.00	
35–39 years	176	51.8%	164	48.2%		1.01	(0.83–1.23)
≥40 years	33	44.0%	42	56.0%		1.13	(0.81–1.57)
Pre-pregnancy BMI, kg/m^2^							
<18.5	150	46.0%	176	54.0%	0.163	1.05	(0.89–1.25)
18.5–24.9	591	50.7%	574	49.3%		1.00	
25–29.9	42	41.2%	60	58.8%		1.16	(0.88–1.51)
≥30	13	46.4%	15	53.6%		1.08	(0.64–1.81)
Parity							
Nulliparous	405	46.7%	463	53.3%	0.034	1.12	(0.90–1.40)
Multiparous	391	51.9%	362	48.1%		1.00	
Use of in vitro fertilization							
Yes	91	46.7%	53.3	53.3%	0.468	1.12	(0.90–1.40)
No	91	49.4%	721	50.6%		1.00	
Marital status							
Married	788	49.6%	802	50.4%	0.009	0.76	(0.49–1.17)
Other	8	25.8%	23	74.2%		1.00	
Alcohol consumption status							
Never	162	44.4%	203	55.6%	0.041	1.00	
Former	597	50.0%	597	50.0%		0.76	(0.51–1.13)
Current	37	59.7%	25	40.3%		0.87	(0.33–2.28)
Active smoking status							
Never	595	49.3%	613	50.7%	0.909	1.00	
Former	186	48.9%	194	51.1%		0.99	(0.83–1.17)
Current	15	45.5%	18	54.5%		1.01	(0.63–1.64)
Daily passive smoking							
Yes	79	46.5%	91	53.5%	0.468	1.07	(0.85–1.34)
No	717	49.4%	734	50.6%		1.00	
Maternal medical complications^a^							
Yes	155	45.6%	185	54.4%	0.144	1.06	(0.90–1.25)
No	641	50.0%	640	50.0%		1.00	
Educational attainment							
≥13 years	698	51.0%	671	49.0%	<0.001	0.87	(0.72–1.05)
<13 years	98	38.9%	154	61.1%		1.00	
Household income per year							
<5 million JPY	226	46.0%	265	54.0%	0.002	1.00	(0.83–1.20)
5 to <8 million JPY	155	43.3%	203	56.7%		1.00	
≥8 million JPY	364	54.7%	302	45.3%		0.89	(0.75–1.05)
Declined to answer or do not know	51	48.1%	55	51.9%		1.00	(0.74–1.34)
Occupational status							
Working	327	52.2%	299	47.8%	0.011	0.87	(0.74–1.04)
Not working with maternal leave	214	51.3%	203	48.7%		0.93	(0.76–1.13)
Not working without maternal leave	255	44.1%	323	55.9%		1.00	
Local state of emergency							
None	109	41.3%	155	58.7%	0.017	1.00	
Semi-emergency	217	52.0%	200	48.0%		0.83	(0.67–1.04)
Emergency	470	50.0%	470	50.0%		0.90	(0.74–1.09)
Trust in the government							
Yes	156	58.6%	110	41.4%	0.001	1.00	
No	640	47.2%	715	52.8%		1.26	(1.03–1.54)
Current or previous COVID-19							
Yes	7	50.0%	7	50.0%	0.946	1.04	(0.48–2.22)
No	789	49.1%	818	50.9%		1.00	
Fear of COVID-19 scale (continuous)						0.99	(0.98–1.01)

aPR, adjusted prevalence ratio; BMI, body mass index; CI, confidence interval; COVID-19, coronavirus disease 2019.

^a^Maternal complications included chronic hypertension, diabetes mellitus, asthma, thyroid disorders, chronic kidney disease, and autoimmune diseases.

Adjusted for all variables in the table.

## DISCUSSION

We used the JACSIS study which covered all prefectures in Japan, and the proportion of pregnant women in every prefecture was not different from government data in 2020.^24^ Among the pregnant women who were surveyed, 13.4% had received at least one COVID-19 vaccination as of July–August 2021. While vaccine hesitancy accounted for 50.9% of the total study population, only 8.4% were obviously avoidant, and 42.5% responded with the option, “wait and see.” The main reasons for vaccine hesitancy were concerns about negative effects on the fetus and breastfed infant, adverse reactions, and the trustworthiness and effectiveness of the vaccine components. Knowledge of the key reasons for vaccine hesitancy among pregnant women is important to improve vaccine dissemination in the future.

The period under study, July 28 to August 30, 2021, was a time of explosive spread of the COVID-19 pandemic in Japan. As a result, the cumulative number of infected people increased from 892,753 on July 28 to 1,476,805 on August 30.^25^ During the survey period, on August 20, the number of new infections was the highest up till then at 25,992 (weekly average: 21,247.4).^25^ As a result, a state of emergency was declared in various prefectures (eTable 2). The situation was such that vaccination of the general population was underway throughout the country, and pregnant women were included in the target population. Regarding the vaccine coverage rate, as of July 2021, 70% of the elderly who were eligible for priority vaccination had received the first dose, while as of July 28, 2021, only 38.6% of the total population had received the first dose. Under these circumstances, we should be cautious while assessing the 13% first dose vaccination rate among pregnant women as revealed in this survey. This is because, first, the survey does not assess whether the pregnant women themselves had the opportunity to be vaccinated and includes those who would have been vaccinated if they had the opportunity. Second, pregnant women may not have received the message from the government and academic societies that the recommendation for COVID-19 vaccination has changed.^11^^,^^12^ The government announced in August 2021 that it would recommend vaccination for all pregnant women, and since then, local governments have started vaccination programs for pregnant women on a priority basis. In light of these factors, we believe that the vaccination rate among pregnant women cannot be considered extremely low. It is important to clarify how many pregnant women are actually being vaccinated under the current situation, where pregnant women can be vaccinated if they wish. We plan to conduct a follow-up survey in 2022 to investigate the answers to these questions.

There are few reports on vaccine aversion to the COVID-19 vaccine in Japan.^14^^,^^15^ Our study is of particular importance because it clarifies the vaccine-related anxiety of pregnant women, for whom vaccination is considered important. Previous studies have shown that the rate of vaccine hesitancy is higher among young people in Japan.^14^^,^^15^ In a February 2021 survey using the same questioning method as this study, Okubo et al reported that 15% of women aged 45 and under answered “I do not want to be vaccinated,”^14^ while in our study, the rate was 8.4%.

The main reasons for vaccine hesitancy reported by this study’s population were related to concerns regarding the effects of the vaccine on the fetus and lactation, as well as the risk of experiencing adverse reactions. Previous studies have not reported elevated pregnancy complications after vaccination, and there have been reports of neutralizing antibodies being transferred to the fetus and breastmilk following vaccination during pregnancy.^6^^–^^8^ By disseminating this information to pregnant women, it may be possible to reduce their vaccine-related anxiety. Since most pregnant women in Japan undergo prenatal checkups at medical institutions, they have more opportunities than the general population to receive information from medical professionals, who have the most current information resources. To ensure that the correct information is provided to pregnant women, medical institutions should actively disseminate information to them, rather than having them look for information themselves. To do this, the government and the Japanese Society of Obstetrics and Gynecology should create a method of disseminating information in a uniform format that is easy to use, and COVID-19 vaccine information can be included in the maternal and child health handbook.

A previous review showed that concerns about vaccine safety are associated with vaccine hesitancy during pregnancy,^26^ but the reasons for vaccine hesitancy differ according to population.^26^ Some studies have reported that education level and employment status are associated with COVID-19 vaccine hesitancy in pregnant women.^27^^,^^28^ In this study, mistrust in the government was associated with increased vaccine hesitancy. The results of some studies similarly showed that a lack of trust in the government was associated with COVID-19 vaccine hesitancy.^14^^,^^29^^–^^31^ Education level and employment status were also assessed; however, while univariate analysis showed the same association with vaccine avoidance as in previous reports, multivariate results were not significant. As for education level, it was difficult to evaluate the similarity between ours and previous results, because the previous reports were from overseas. Employment status may not have been significant in previous reports because the categorization took maternity leave into account.

There are several limitations to this study. First, due to the study’s cross-sectional design, it was not possible to prove causality. Second, this was an Internet survey and, hence, subject to selection bias. However, pregnant women are a relatively younger generation among the general population; it is a commonly held conviction that few populations in Japan do not have access to the Internet. Third, for women near their due dates, “I want to be vaccinated” may include women who are hesitant to get vaccinated while pregnant and intend to be vaccinated after delivery. Fourth, we were not able to investigate whether the participants had the opportunity to receive the COVID-19 vaccination in this survey. As a result, it was not possible to indicate the vaccination rate in the population of pregnant women who had the opportunity to be vaccinated. Fifth, some participants may have given socially acceptable answers; this effect might result in a reduction in the rate of vaccine hesitancy.

To the best of our knowledge, this is the first study on vaccine hesitancy among pregnant women in Japan. Pregnancy places women at an increased risk of severe COVID-19 infection; therefore, prevention of COVID-19 infection in pregnant women is essential. Efforts should be made to promote COVID-19 vaccination during pregnancy. The reasons for vaccine hesitancy and factors associated with avoidance identified in this study will be useful in developing strategies to promote COVID-19 vaccination for pregnant women.

In conclusion, there is currently insufficient COVID-19 vaccination of pregnant women in Japan. It is necessary to expand the Japanese vaccination system and disseminate accurate and current information about the safety and efficacy of COVID-19 vaccination during pregnancy to minimize vaccine hesitancy in pregnant women.

## APPENDIX A. SUPPLEMENTARY DATA

eTable 1. Prefecture distribution of participants and all Japanese women between the ages of 20 and 50 years old

eTable 2. Trends in the declaration of a state of emergency and semi-emergency in prefectures in Japan during the survey period

eTable 3. The reasons for vaccine hesitancy and acceptance among all pregnant women in the study population
