# Supplementary material for: The Prevalence of COVID-19 Vaccination and Vaccine Hesitancy in Pregnant Women: An Internet-based Cross-sectional Study in Japan
Source: J Epidemiol. 2022 Apr 5;32(4):188–94. doi: 10.2188/jea.JE20210458 (PMC8918615; doi:10.2188/jea.JE20210458)
Supplement: Supplementary file 1 [file je-32-188-s001.pdf]

eTable 1. Prefecture distribution of participants and all Japanese women between the ages of 20 and 50 years old

	All pregnant women n=1,621		Women between the ages of 20 and 50 years old[a] n=21,385,480	
	N	%	N	%
Hokkaido	69	4.3%	852,270	4.0%
Aomori	12	0.7%	186,850	0.9%
Iwate	15	0.9%	180,134	0.8%
Miyagi	26	1.6%	395,843	1.9%
Akita	8	0.5%	133,611	0.6%
Yamagata	8	0.5%	157,451	0.7%
Fukushima	13	0.8%	283,832	1.3%
Ibaraki	28	1.7%	457,670	2.1%
Tochigi	13	0.8%	311,780	1.5%
Gunma	14	0.9%	303,765	1.4%
Saitama	84	5.2%	1,278,916	6.0%
Chiba	86	5.3%	1,079,368	5.0%
Tokyo	215	13.3%	2,732,942	12.8%
Kanagawa	141	8.7%	1,637,391	7.7%
Niigata	23	1.4%	339,664	1.6%
Toyama	17	1.0%	160,076	0.7%
Ishikawa	10	0.6%	185,127	0.9%
Fukui	10	0.6%	119,996	0.6%
Yamanashi	4	0.2%	124,586	0.6%
Nagano	21	1.3%	314,660	1.5%
Gifu	19	1.2%	311,264	1.5%
Shizuoka	33	2.0%	571,764	2.7%
Aichi	136	8.4%	1,296,231	6.1%
Mie	24	1.5%	280,724	1.3%
Shiga	22	1.4%	241,726	1.1%
Kyoto	42	2.6%	433,516	2.0%
Osaka	143	8.8%	1,592,330	7.4%

Hyogo	73	4.5%	940,222	4.4%
Nara	18	1.1%	220,184	1.0%
Wakayama	13	0.8%	145,974	0.7%
Tottori	3	0.2%	85,885	0.4%
Shimane	11	0.7%	97,532	0.5%
Okayama	16	1.0%	311,301	1.5%
Hiroshima	52	3.2%	462,122	2.2%
Yamaguchi	15	0.9%	201,415	0.9%
Tokushima	3	0.2%	112,767	0.5%
Kagawa	14	0.9%	155,547	0.7%
Hiroshima	10	0.6%	210,105	1.0%
Kochi	2	0.1%	104,624	0.5%
Fukuoka	66	4.1%	911,395	4.3%
Saga	10	0.6%	130,220	0.6%
Nagasaki	13	0.8%	201,256	0.9%
Kumamoto	26	1.6%	275,514	1.3%
Oita	9	0.6%	174,646	0.8%
Miyazaki	5	0.3%	166,417	0.8%
Kagoshima	15	0.9%	248,916	1.2%
Okinawa	11	0.7%	265,951	1.2%

[a] Women aged 20 to 50 years old by prefecture were extracted from government data[1].

eTable 2. Trends in the declaration of a state of emergency and semi-emergency in prefectures in Japan during the survey period

State of emergency	
05/23/2021	Okinawa
07/12/2021	Tokyo
08/02/2021	Saitama, Chiba, Kanagawa, Osaka
08/20/2021	Ibaraki, Tochigi, Gunma, Shizuoka, Kyoto, Hyogo, Fukuoka
08/27/2021	Hokkaido, Miyagi, Gifu, Aichi, Mie, Shiga, Okayama, Hiroshima

State of semi emergency	
08/02/2021	Hokkaido (08/26), Ishikawa, Kyoto (08/19), Hyogo (08/19), Fukuoka (08/19)
08/08/2021	Fukushima, Ibaraki (08/19), Tochigi (08/19), Gunma (08/19), Shizuoka (08/19), Aichi (08/26), Shiga (08/26), Kumamoto
08/20/2021	Miyagi (08/26), Toyama, Yamanashi, Gifu (08/26), Mie (08/26), Okayama (08/26), Hiroshima (08/26), Kagawa, Ehime, Kagoshima
08/27/2021	Kochi, Saga, Nagasaki, Miyazaki

All declarations were still in place on August 30, 2021, at the end of the survey period.

eTable 3. The reasons for vaccine hesitancy and acceptance among all pregnant women in the study population

	Already been vaccinated n=217	Want to be vaccinated n=579	Want to 'wait and see' before getting the vaccine n=689	Do not want to be vaccinated n=136
I do not have time to get vaccinated	-	-	114 (16.5)	13 (9.6)
I am worried about adverse reactions	-	-	576 (83.6)	113 (83.1)
I am concerned about the potential effects on my fetus	-	-	588 (85.3)	120 (88.2)
I am concerned about the potential effect on lactation	-	-	466 (67.6)	94 (69.1)
I do not think it is very effective	-	-	146 (21.2)	60 (44.1)
I do not think it is very effective in preventing severe symptoms	-	-	82 (11.9)	42 (30.9)
I do not trust the components of vaccines	-	-	338 (49.1)	111 (81.6)
I do not think I will get infected	-	-	22 (3.2)	14 (10.3)
I think I have a low risk of getting seriously ill	-	-	45 (6.5)	18 (13.2)
It was recommended by a family member or friend	84 (38.7)	231 (39.9)	103 (14.9)	23 (16.9)
It was recommended by SNS or the media	66 (30.4)	195 (33.7)	59 (8.6)	5 (3.7)
I am worried about getting infected with COVID-19	195 (89.9)	551 (95.2)	343 (49.8)	-
I think I have a high risk of becoming seriously ill	140 (64.5)	288 (49.7)	149 (21.6)	-
I am a medical worker	61 (28.1)	70 (12.1)	48 (7.0)	-

I do not want to infect my family or other people around me	193 (88.9)	534 (92.2)	402 (58.3)	-
I think it is necessary for society to be vaccinated	173 (79.7)	443 (76.5)	257 (37.3)	-
I am more worried about getting a severe infection than I am about the risk of adverse reactions	189 (87.1)	493 (85.1)	270 (39.2)	-

Values are given as frequency (percentage).

COVID-19, coronavirus disease 2019; SNS, social networking service.

REFERENCES

1. Population, demographics, and number of households based on the Basic Resident Ledger. https://www.soumu.go.jp/main_sosiki/jichi_gyousei/daityo/jinkou_jinkoudoutai-setaisuu.html. 2021. Accessed 11.26.2021.
